# Unveiling the Unseen Struggles: A Comprehensive Review of Vitiligo's Psychological, Social, and Quality of Life Impacts

**DOI:** 10.7759/cureus.45030

**Published:** 2023-09-11

**Authors:** Abdelaziz H Salama, Lujain Alnemr, Ahmad R. Khan, Hussein Alfakeer, Zoha Aleem, Mohamed Ali-Alkhateeb

**Affiliations:** 1 Medical School, Hamidiye International School of Medicine, University of Health Sciences, Istanbul, TUR; 2 Internal Medicine, University Hospital Limerick, Limerick , IRL; 3 Internal Medicine, Batterjee Medical College, Jeddah, SAU

**Keywords:** self-esteem, social stigma, psychology, depigmentation, depression, anxiety, autoimmune disorder, loss of skin pigmentation

## Abstract

This review explores the psychosocial impact of vitiligo on patients, its consequences for their quality of life, and the need for holistic support.

Vitiligo's psychosocial burden, driven by the need to conceal lesions and societal beauty ideals, leads to stress, sadness, and low self-esteem. Social stigma affects self-esteem, especially in cultural contexts, exacerbating the need for culturally sensitive support. Anxiety and depression are common due to visible differences and societal pressures.

Vitiligo significantly reduces the quality of life, especially in younger patients, impacting daily activities, careers, and relationships. Disease severity worsens these effects, particularly in visible areas and among individuals with darker skin tones. Long-term disease activity may improve acceptance and quality of life.

Psychological support and counseling are crucial, as many patients don't seek medical help. Education plays a key role, improving understanding and reducing anxiety. Raising awareness about the impact of vitiligo can challenge perceptions and contribute to enhancing patients' well-being.

In conclusion, this review highlights the interplay between psychosocial factors, quality of life, and the importance of addressing social stigma, providing psychological support, and advancing education and awareness for those with vitiligo.

## Introduction and background

Vitiligo, an acquired disorder resulting in the loss of skin pigmentation, is a chronic condition of depigmentation with global significance. Its estimated prevalence ranges around 1-2% worldwide, though precise figures can be challenging to ascertain (Table 1) [1]. Recent advancements have significantly enhanced our comprehension of vitiligo's origin, now definitively categorized as an autoimmune disorder [2]. This disorder involves a targeted reduction in melanocytes, subsequently leading to the depigmentation of specific skin areas. It's important to note that vitiligo presents as well-defined, fully depigmented, chalky-white patches [3]. Despite its wide occurrence, a conclusive cure for vitiligo remains elusive, and the efficacy of available treatments varies individually, frequently yielding unsatisfactory outcomes [1].

**Table 1:** Overview of Vitiligo Prevalence Studies

	Prevalence type	Description	Range/Percentage	Notes	Ref.
1	Global Prevalence	Estimated worldwide prevalence	0.5–1%	It is difficult to estimate precisely.	[15]
2	Bornholm Study	Prevalence on the island of Bornholm in Denmark	0.38%	A most extensive epidemiological study in 1977 and One of the earliest surveys in 1977.	[16]
3	French West Indies	Prevalence in the black population of the French West Indies	Similar to or slightly less than the white population.		[17]
4	Regional Prevalence	The highest incidence: India, followed by Mexico and Japan	India: up to 8.8%, Mexico: 2.6–4%, Japan: 1.68%	Disparity between prevalence and incidence data.	[18]
5	Gender Differences	Equal prevalence in males and females		Gender-related seeking of consultation.	[14,19]
6	Meta-Analysis	Pooled prevalence from various studies	Community-based: 0.2%, Hospital-based: 1.8%	from 82 population- or community-based studies and 22 hospital-based studies.	[20]
7	Age-Related Prevalence	Vitiligo prevalence and age distribution		Non-segmental vitiligo occurs across ages; segmental vitiligo tends to appear young.	[21]
8	Age-Related Increase	Vitiligo prevalence increases with age	Varies with different age groups.	Supported by multiple studies.	[22,23]
9	Multinational Study (Europe, USA, and Japan)	vitiligo prevalence among 35,694 survey participants (Europe, n = 18 785; USA, n = 8517; Japan, n = 8392)	1.3%	The overall prevalence is 1.3%, with 0.6% diagnosed cases, 0.4% undiagnosed cases, and 0.3% displaying signs of vitiligo.	[24]
10	Korean Study	Three-year period, comprehensive review using Korean population data	0.12–0.13%	Vitiligo in the Korean population is associated with various autoimmune/non-autoimmune diseases, including thyroiditis, atopic dermatitis, and psoriasis.	[25]

While the skin is the main area affected by vitiligo, its effects reach beyond the apparent. Numerous psychosocial difficulties are frequently encountered by those who have vitiligo [4].

The impact of vitiligo on a person's psychological and social well-being extends to various facets of life, encompassing emotional health, social interactions, and opportunities in employment. The emergence of white patches on the skin can trigger a complex range of emotional reactions, such as nervousness [5], decreased self-confidence [6], anxiety, and even depression [7]. These emotional responses can significantly diminish an individual's overall quality of life [8], impacting the person and their close circle. The study conducted by Bin Saif et al. (2013) highlights the significant impact of vitiligo on the quality of life (QoL) of family members and close relatives. Their research found that 91.5% of subjects experienced QoL effects due to their association with vitiligo patients [9]. Additionally, the severe psychosocial effects of vitiligo may include feelings of paranoia [10] and embarrassment [11], which can significantly interrupt everyday activities. The impact can extend to sleep disturbances [12] and even interfere with intimate relationships, negatively influencing sexual relations [13]. In some cases, vitiligo has been found to negatively impact marital relationships, with instances where the emergence of skin depigmentation served as grounds for divorce [14]. This combination of emotional and social challenges underscores the importance of addressing vitiligo's impact comprehensively.

The primary aim of this study is to underscore the importance of tailored support mechanisms and interventions that address the emotional well-being and career goals of individuals living with vitiligo. This study aims to highlight the need for specialized support by addressing the complex psychosocial aspects of the disease. Such an approach can contribute to creating more inclusive environments where individuals have a fair opportunity to succeed, regardless of their dermatological condition.

## Review

Psychosocial impact of vitiligo

Vitiligo patients had an ongoing burden due to the unpredictable onset of the condition and the need to cover it up with clothing or makeup [26]. Patients who reported that they thought about their condition constantly throughout the day and became self-conscious while looking in the mirror, even with the lesions covered, are examples of the strong psychosocial impact [27]. This low self-perception, which frequently resulted in stress, sadness, and low self-esteem, was caused by their unhappiness with their appearance [27]. While vitiligo doesn't pose a challenging physical obstacle, its impact on a patient's well-being and quality of life is considerable and unrelated to the illness's severity [28-30].

Social Stigma and Self-Esteem

The psychosocial impact of vitiligo is underscored by the intricate interplay between visible differences and social stigma, which significantly affects an individual's self-esteem. Thompson et al. (2010), conducted within the context of British South Asian women, delve into the experiences of living with vitiligo [26]. The study emphasizes that societal perceptions, cultural norms, and beauty ideals are pivotal in shaping the emotional landscape of individuals with vitiligo.

Thompson et al. (2010) shed light on the challenges faced by individuals with vitiligo, especially within the British South Asian community, where they confront adverse reactions, misconceptions related to the disease, and discriminatory attitudes owing to their altered appearance [26]. They find resonance in the work of Ezzedine et al. (2021) [4]**.** Ezzedine et al.'s study delves into the psychosocial effects of vitiligo through a systematic literature review, providing a broader perspective on how individuals with vitiligo often grapple with social stigma and prejudice stemming from misunderstandings and societal beauty standards [4]. The common thread among these studies underscores the universal nature of social bias and its potential to impact an individual's self-esteem adversely.

It is noteworthy that studies like Thompson et al. (2010) and Ezzedine et al. (2021) indicate that this stigma contributes to lowered self-esteem and diminished confidence among individuals with vitiligo [26,4]. The societal bias these individuals face may lead to a negative self-perception, impacting how they view themselves and their place in society. Furthermore, within specific cultural contexts, the influence of unique cultural norms and societal beauty ideals becomes more pronounced, intensifying the pressure on individuals to adhere to established appearance standards. This increased emphasis on conformity to cultural expectations can further compound the marginalization experienced by those affected by vitiligo. Therefore, there is a growing urgency to establish support systems that are culturally sensitive and tailored to address these unique challenges effectively. 

Anxiety and Depression

The psychosocial implications of vitiligo extend to mental health, with anxiety and depression being significant concerns. Kussainova et al. (2020) conducted a systematic review and meta-analysis that emphasized the link between vitiligo and anxiety. Their study reported a general prevalence of anxiety among vitiligo patients equal to 35.8% [31]. Similarly, Lai et al. (2017) conducted a systematic review and meta-analysis focusing on vitiligo's association with depression. Their study revealed that patients with vitiligo were at a significantly higher risk of clinical depression or depressive symptoms compared to those without a depigmenting disease. Approximately one-third of patients with vitiligo reported depressive symptoms or impaired general health, and up to one-quarter of them had clinical depression [32].

The significance of these studies lies in highlighting the emotional toll of vitiligo, emphasizing that the visible nature of the condition amplifies the risk of anxiety and depression. Recognizing this, interventions that address the physical and emotional dimensions of vitiligo become imperative. By shedding light on these connections, the studies by Kussainova et al. (2020) and Lai et al. (2017) underscore the necessity of holistic approaches to support individuals navigating the challenges posed by vitiligo [31,32].

Quality of life of patients with vitiligo

Quality of life (QoL) is defined, according to the World Health Organization, as a subjective assessment of how effectively one's reality aligns with one's goals as seen through a perspective of one's culture and value system. Consequently, quality of life encompasses elements such as relationships, educational objectives, employment status, workplace atmosphere, and social position [33]. Considering the skin's status as the body's largest and most prominent organ, diseases affecting it can impact an individual's quality of life negatively [34]. Despite vitiligo being frequently thought of as a cosmetic disorder, vitiligo has been identified as a significant factor leading to a considerable reduction in quality of life, especially in younger patients [35-38].

Impact on Daily Life and Activities

Vitiligo has an adverse effect on several quality-of-life aspects, including self-consciousness, socializing and leisure activities, working or studying [39]. It is shown that vitiligo influences career choices, leading to the denial of certain job opportunities for patients [27]. The severity of the disease is correlated with the overall quality of life, with more negative impacts on quality of life often associated with greater involvement of body surface area and greater visibility of lesions [40]. Studies have demonstrated that patients' quality of life is adversely affected by vitiligo lesions that are present on visible areas such as the hands or the face or sensitive areas such as the genitalia [41, 42]. The appearance of vitiligo can have significant consequences on the quality of life for individuals with darker skin, attributed to the noticeable contrast [29]. However, patients who had experienced disease activity for more than a decade exhibited a higher quality of life. This increase in quality of life may be potentially linked to the prolonged acceptance of vitiligo over an extended period [24,43].

Relationships and Social Support

The pigment-related distortions resulting from vitiligo can significantly impact social interactions [44]. Vitiligo patients often undergo emotional challenges that can result in social avoidance and coping strategies for managing the condition include wearing concealing clothing and using methods to camouflage the affected areas [26,45]. Vitiligo exerts an adverse effect on both the personal lives and marital status of patients, which in turn is reflected in the quality of life [46]. Individuals with vitiligo often experience distress and feelings of embarrassment in the context of initiating sexual relationships and emotional connections [46]. In comparison to unmarried patients with vitiligo, those who were married demonstrated more severe emotional and appearance-related disruptions [47, 48]. In contrast to men, female vitiligo patients experienced more significant impairments in general and psychological health, intimate relationships, and sexual experiences [49]. Among females with genital vitiligo, sexual function was notably more compromised compared to those without genital lesions or lesions on other parts of the body [50]. Furthermore, vitiligo leads to emotional distress and diminishes the quality of life for both patients and their parents during childhood [51]. Among parents or caregivers of children with pediatric vitiligo, reports indicate that 26% experienced depression, while 42% experienced anxiety [52].

Psychological support and counseling

Recent findings reveal a notable disparity between the prevalence of vitiligo as indicated by primary clinical data and the number of medical claims related to vitiligo treatment. This discrepancy suggests that a significant number of individuals with vitiligo do not actively seek medical intervention even after facing years of frustrating treatment attempts [53, 54]. Although there is a clear indication that individuals with vitiligo could be vulnerable to developing social anxiety, only a few studies have tried to explore strategies for reducing this distress [55].

Education and Awareness

Research indicates that a correlation exists between higher levels of education and a greater ability among patients to comprehend the disease rationally, consequently reducing the burden caused by the condition [56]. The initiation of psychological intervention for treating vitiligo involves educating patients about their treatment. Improving knowledge about the condition and its standardized treatment can enhance patients' understanding of the disease, significantly reduce anxiety and fear, and promote a greater determination to address the condition actively [57]. Certain individuals might decide not to seek a formal diagnosis due to their personal beliefs and religions that vitiligo is untreatable or heavily influenced by their behavior [58].

In a recent study investigating Health-Related Quality of Life (HRQoL) about prevalent medical conditions, it was discovered that patients with higher educational levels exhibited a notably elevated HRQoL. [59]. Raising awareness about the quality-of-life challenges linked to vitiligo, coupled with enhanced insights into vitiligo's underlying causes and its connections to systemic conditions, will contribute to challenging the perception of vitiligo as a cosmetic or insignificant condition [60].

Additionally, it is worth highlighting that famous individuals who have openly dealt with vitiligo, such as Winnie Harlow, Michael Jackson, Lee Thomas, and Big Krizz Kaliko, have played a significant role in raising awareness and promoting education about the condition. These influential figures serve as inspiring role models and exemplify that vitiligo need not hinder one's pursuit of success and fulfillment. Their stories underscore the importance of psychological support, self-acceptance, and the potential for individuals with vitiligo to lead fulfilling lives despite the challenges posed by the condition [61].

## Conclusions

In conclusion, this review highlights the complex impact of vitiligo on individuals' lives, extending beyond its physical appearance. The visible nature of the condition intensifies emotional distress, affecting self-esteem and confidence. Disruptions in daily life and relationships underscore the need for tailored interventions.

A comprehensive approach involving education, awareness campaigns, and psychological support is essential. Collaborative efforts can create a more inclusive and empathetic environment, addressing the emotional and psychosocial challenges of vitiligo and enhancing well-being.
